# Sampling from commercial vessel routes can capture marine biodiversity distributions effectively

**DOI:** 10.1002/ece3.9810

**Published:** 2023-02-11

**Authors:** Elizabeth Boyse, Maria Beger, Elena Valsecchi, Simon J. Goodman

**Affiliations:** ^1^ School of Biology University of Leeds Leeds UK; ^2^ Department of Environmental and Earth Sciences University of Milano‐Bicocca Milan Italy

**Keywords:** biased sampling frames, cetaceans, environmental DNA, ferries, marine megafauna, species distribution models

## Abstract

Collecting fine‐scale occurrence data for marine species across large spatial scales is logistically challenging but is important to determine species distributions and for conservation planning. Inaccurate descriptions of species ranges could result in designating protected areas with inappropriate locations or boundaries. Optimizing sampling strategies therefore is a priority for scaling up survey approaches using tools such as environmental DNA (eDNA) to capture species distributions. In a marine context, commercial vessels, such as ferries, could provide sampling platforms allowing access to undersampled areas and repeatable sampling over time to track community changes. However, sample collection from commercial vessels could be biased and may not represent biological and environmental variability. Here, we evaluate whether sampling along Mediterranean ferry routes can yield unbiased biodiversity survey outcomes, based on perfect knowledge from a stacked species distribution model (SSDM) of marine megafauna derived from online data repositories. Simulations to allocate sampling point locations were carried out representing different sampling strategies (random vs regular), frames (ferry routes vs unconstrained), and number of sampling points. SSDMs were remade from different sampling simulations and compared with the “perfect knowledge” SSDM to quantify the bias associated with different sampling strategies. Ferry routes detected more species and were able to recover known patterns in species richness at smaller sample sizes better than unconstrained sampling points. However, to minimize potential bias, ferry routes should be chosen to cover the variability in species composition and its environmental predictors in the SSDMs. The workflow presented here can be used to design effective sampling strategies using commercial vessel routes globally for eDNA and other biodiversity survey techniques. This approach has potential to provide a cost‐effective method to access remote oceanic areas on a regular basis and can recover meaningful data on spatiotemporal biodiversity patterns.

## Graphical abstract

Commercial vessels as sampling platforms can accurately capture species distribution patterns, despite inherent biases associated with constrained spatial coverage. Our workflow can be applied across the global shipping network to upscale sampling using techniques such as environmental DNA to reduce gaps in marine biodiversity knowledge.

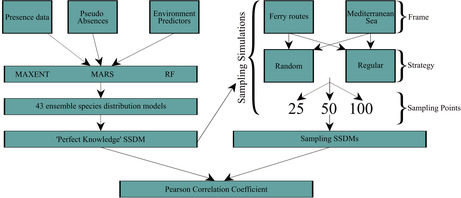

## 1 INTRODUCTION

Knowledge of species' ranges is essential for assessments of conservation status, to detect changes in distributions, and to inform spatial planning decisions (Wetzel et al., 2018). Initiatives to aggregate biodiversity data, including the Global Biodiversity Information Facility (GBIF) and the Ocean Biodiversity Information System (OBIS), have increased access to global standardized datasets (Grassle, 2000; Telenius, 2011). However, these datasets are limited by data quality issues, such as positional accuracy or duplicates of records, and spatial, temporal, and taxonomic biases (Moudrý & Devillers, 2020). Marine species and habitats are underrepresented due to the monetary and logistical challenges of collecting data, with up to 50% of records for marine taxa being collected from coastal regions or are classified as Data Deficient in IUCN Red List assessments (Dulvy et al., 2014; Hughes et al., 2021). Data limitations increase uncertainty in marine spatial planning prioritisations and could lead to less efficient marine reserve systems (Bani et al., 2020; Foley et al., 2010). Novel methods that provide high‐quality biodiversity data are needed for remote areas to improve our knowledge of species distributions, and their conservation. This paper presents a novel framework to design sampling strategies using commercial vessels as data collection platforms that could help to scale up surveys to record species communities more accurately and comprehensively.

In biodiversity surveys, it is usually infeasible to collect samples at very high coverage across large geographical scales, so sampling strategies target the collection of non‐biased data at resolutions relevant to the study aims. Design‐based sampling methods, including random, regular, and stratified random sampling, ensure that every sampling unit has a non‐zero probability of being sampled (Wang et al., 2012). Model‐based sampling designs aim to avoid bias by considering spatial autocorrelation and heterogeneity in the sampling frame, the area to which sampling is restricted (Zhang et al., 2020). The choice of sampling design is dependent on the study objectives and study area characteristics as no method consistently outperforms others (Zhang et al., 2020). These sampling designs assume that it is possible to access the entire sampling frame for sample collection. However, in the marine environment, this is often impossible to achieve, especially when considering the large spatial scales relevant for marine spatial planning, or the conservation of highly mobile species (Notarbartolo di Sciara et al., 2016).

Commercial vessels, such as ferries, typically follow specific shipping routes covering large spatial scales comprehensively, making them effective platforms for replicable sampling transects. Ferry‐based sampling is a similar concept to collecting samples close to road networks, which is commonly employed in terrestrial biodiversity surveys due to greater accessibility (Kadmon et al., 2004). The data collected can be biased because the presence of roads directly affects species distributions or because they do not represent the environmental gradients in the whole sampling frame (Kadmon et al., 2004). We therefore need to explore sampling methods that can best capture variability in species distributions from restricted sampling frames, as these often offer us low‐cost sampling and accessibility to hard‐to‐reach areas. Samples from a restricted area (i.e., road networks or shipping routes) can still produce species distribution model predictions similar to samples collected from an unconstrained area if the environmental gradients are adequately captured (Tessarolo et al., 2014). For commercial vessel surveys to be effective, we need a framework for selecting networks of individual routes to accurately capture species composition, for which there is no precedent despite their frequent implementation in visual cetacean surveys and continuous marine plankton recorder surveys (Arcangeli et al., 2017; Reid et al., 2003). Furthermore, other survey technologies, such as environmental DNA (eDNA) or trawl deployment for fishery surveys, also require effective methods for allocating sample points along ferry routes (Aubert et al., 2018; Valsecchi et al., 2021). Understanding which sampling strategies will reduce the inherent bias of restricted sampling frames will allow us to best leverage these low‐cost sampling opportunities.

Species distribution models can serve as sampling backgrounds for simulating sampling strategies (Tessarolo et al., 2014). Individual species distribution models can be summed using probability or binary predictions to create a stacked species distribution model (SSDM) that predicts species richness (Calabrese et al., 2014). Species distribution models only consider environmental constraints on species distributions, which can lead to overprediction of species richness when combining multiple models, as biotic mechanisms such as dispersal limitations or resource competition are not accounted for (Gavish et al., 2017). However, using stacking methods based on occurrence probabilities instead of thresholding occurrence probabilities leads to SSDMs which predict species richness similarly to macroecological models, whilst also retaining information on individual species (Calabrese et al., 2014; Distler et al., 2015; Grenié et al., 2020). The use of empirical versus simulated communities allows for complex community “organization” to be included in sampling simulations and can highlight areas of important conservation interest, i.e., rare species distribution ranges or gradients of diversity (Miller, 2014). We can use the outputs from SSDMs as a benchmark to assess sampling biases associated with different sampling strategies (Braunisch & Suchant, 2010).

This study develops a novel approach for assessing the suitability of different sampling strategies to reduce biases associated with spatially constrained sampling platforms, such as commercial vessel routes. Such a strategy could be used to gain high‐quality data from pelagic areas that are currently undersampled due to accessibility and monetary constraints (Hughes et al., 2021). Firstly, we quantify the magnitude of bias of a spatially constrained network of ferry routes, relative to unconstrained sampling across the Mediterranean Sea, employing different sampling strategies to allocate sampling points. Second, we consider how environmental variability or species composition impacts the effectiveness of ferry routes as a sampling frame with different subsets of ferry routes. Finally, we evaluate the impact of taxonomic sampling biases on correctly predicting gradients in biodiversity as these biases are pervasive in sampling methods such as eDNA metabarcoding. We use ferry routes in the Mediterranean Sea, but the workflow could be applied to shipping networks anywhere, with any kind of vessel.

## 2 METHODS

### 2.1 Building stacked species distribution models

We assembled a SSDM to represent true species distributions based on observational data from online biodiversity repositories and environmental data. An initial literature search identified 171 species of large marine predators (elasmobranchs, mammals, teleost fish, and turtles) with known occurrences in the Mediterranean Sea. We defined a predator based on two criteria; maximum length greater than or equal to 100 cm and a trophic level greater than or equal to four as reported in FishBase (https://www.fishbase.se/) or SeaLifeBase (https://www.sealifebase.ca/). Nine species were retained that only satisfied one of the criteria (Appendix A; Table A1). Occurrence records for species were downloaded from GBIF (https://www.gbif.org, June 2020, GBIF Occurrence Download https://doi.org/10.15468/dd.tqx2he), OBIS (https://obis.org/) and EurOBIS (https://www.eurobis.org/) and supplemented by the Mediterranean Large Elasmobranchs Monitoring (Medlem) database and ACCOBAMS dataset for elasmobranchs and cetaceans, respectively (ACCOBAMS Survey Initiative, 2020; Mancusi et al., 2020). We subset occurrences to include records from 2000 onwards to correspond with the years that environmental variables were available. We removed occurrence records where GPS coordinates had fewer than three decimal places to improve positional accuracy, and duplicates between the datasets based on species, coordinates, year, and month (Moudrý & Devillers, 2020). Records that had the same species, year, and month but different coordinates as a result of potential rounding between databases were also assumed to be duplicates and removed manually. After quality checking, we only retained species with 40 or more occurrence records to improve model accuracy, leading to 43 species in the final presence‐only dataset, with records for individual species ranging from 41 to 7822 occurrences (Meynard et al., 2019). The selected species were representative of all marine vertebrates including teleost fish (*n* = 20), elasmobranchs (*n* = 13), marine mammals (*n* = 9), and one sea turtle species (Appendix A; Table A1). To account for sampling bias in data repositories, occurrences were spatially thinned with a nearest neighbor distance of 10 km using the spThin R package (Aiello‐Lammens et al., 2015). This approach prevents clusters of occurrences although does not account for large‐scale spatial biases. This method resulted in less than 40 occurrences for ten species, in which case the original data were used instead. We downloaded six environmental predictors, bathymetry, sea surface temperature mean, sea surface temperature range and chlorophyll *a* mean from Bio‐ORACLE, and bathymetric slope and distance from shore from Marspec, in WGS84 projection at a resolution of 0.83 × 0.83 degrees (Assis et al., 2018; Sbrocco & Barber, 2013). These environmental variables are of known importance to marine predators, or their prey species (Azzellino et al., 2012; Klippel et al., 2016; Lambert et al., 2017). These variables were normalized to between 0 and 1 to account for units differing by orders of magnitude.

**FIGURE 1:**
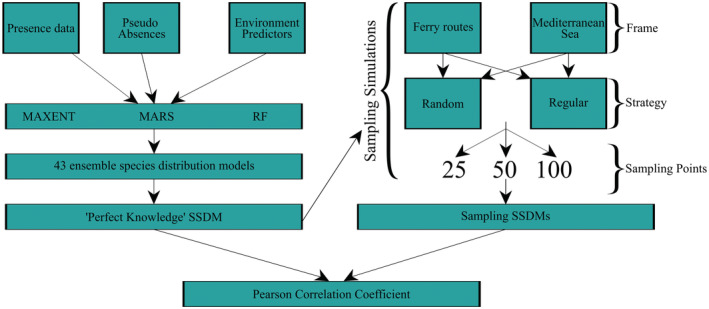
Schematic diagram showing the workflow to create the “perfect knowledge” SSDM using occurrence data from online repositories and extracting occurrence data from the “perfect knowledge” SSDM to build the sampling SSDMs. Sampling SSDMs were compared with the “perfect knowledge” SSDM to evaluate their predictive capacity.

We modeled individual species distributions with three different approaches, maximum entropy (MAXENT), multiple adaptive regression splices (MARS), and random forest (RF). MAXENT was run with 10,000 random background points using the dismo R package (Hijmans et al., 2017). We selected presence‐absence algorithms MARS and RF, despite having a presence‐only dataset as they perform better than presence‐only models when employed with pseudo‐absence data (Barbet‐Massin et al., 2012; Zhang et al., 2019). We generated 1000 pseudo‐absences for MARS and an equal number of pseudo‐absences as presences for RF, both randomly selected within a restricted sampling frame using the two‐degree method as recommended by Barbet‐Massin et al. (2012). We allowed first‐order interactions to be fitted for MARS (Wisz et al., 2008). RF was run with 5000 regression trees and a terminal node of 5 (Zhang et al., 2019). We randomly assigned the dataset into training (70%) and testing (30%) sets three times for cross‐validation (Arenas‐Castro et al., 2022; Sundaram & Leslie, 2021). We assembled the model projections across the three modeling methods using weighted AUC scores for each species. Probabilities of occurrence were translated to binary occurrences using the sensitivity (i.e., true positive rate) equals specificity (i.e., true negative rate) threshold (Liu et al., 2005). The individual species binary ensemble models were then summed to show species richness in the final binary SSDM (Figure 1). We selected a binary SSDM as binary data were required for sampling simulations. This initial SSDM created with occurrence data from online repositories will be referred to as the “perfect knowledge” SSDM for sampling simulation comparisons. All species distribution modeling was carried out using the SSDM R Package using R version 4.1.0 (R Core Team, 2021; Schmitt et al., 2017).

### 2.2 Sampling strategy simulations

**FIGURE 2:**
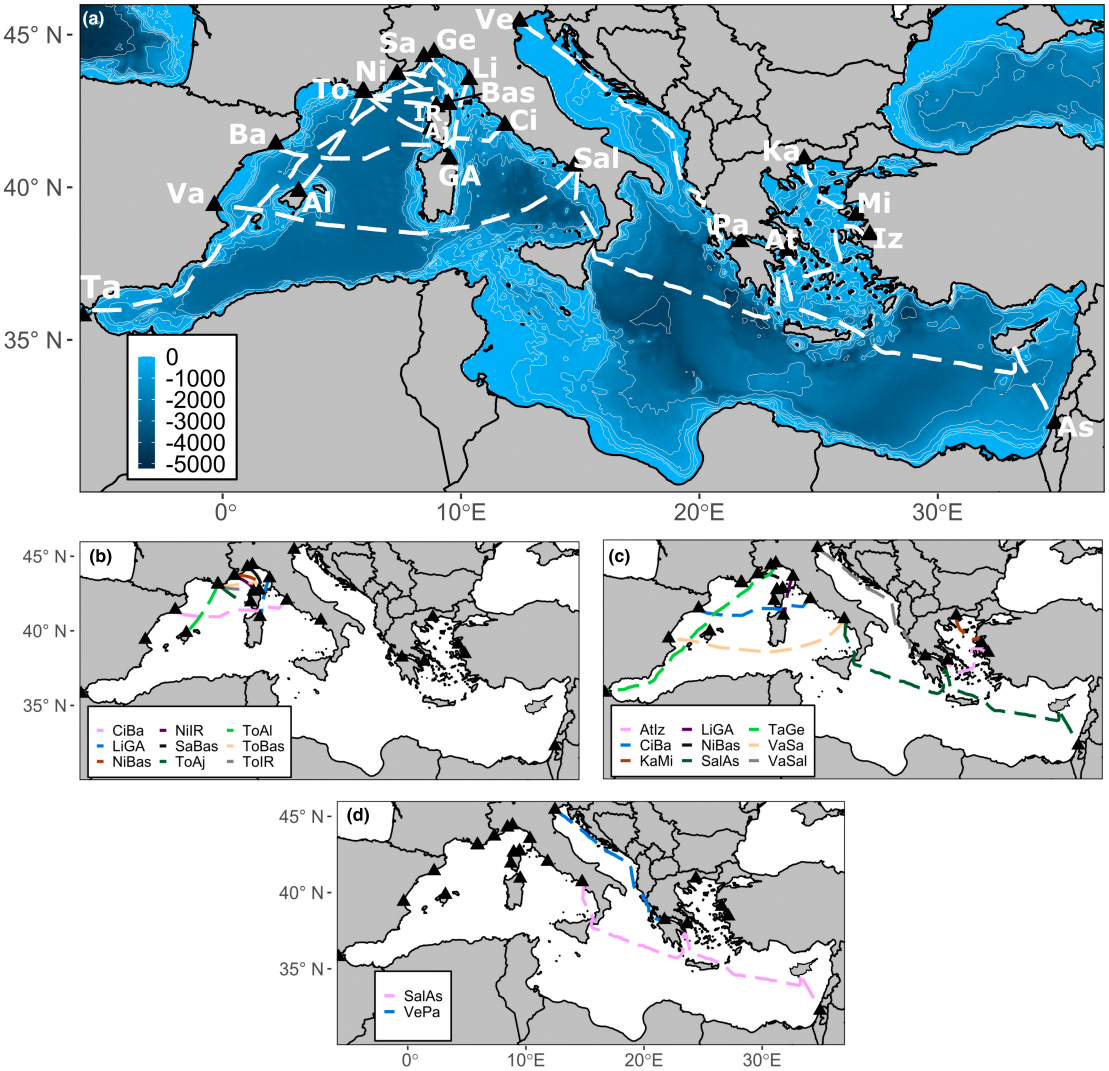
Maps showing the layout of the (a) whole ferry route network consisting of 15 individual ferry routes. Abbreviations for Ports: Aj, Ajaccio; Al, Alcudia; As, Ashdod; At, Athens; Ba, Barcelona; Bas, Bastia; Ci, Civitavecchia; GA, Golfo Aranci; Ge, Genoa; IR, Ille Rousse; Iz, Izmir; Ka, Kavala; Li, Livorno; Mi, Mitilini; Ni, Nice; Pa, Patras; Sa, Savona; Sal, Salerno; Ta, Tangier; To, Toulon; Va, Valencia; Ve, Venice. (b) Biased ferry route subnetwork, (c) community ferry route subnetwork, (d) environment ferry subnetwork.

To enable comparisons of different sampling strategies relative to the “perfect knowledge” SSDM, we selected fifteen operational ferry routes of varying lengths (both intra/inter‐country tracks) to represent the distribution of ferry routes in the Mediterranean Sea (Figure 2a). We simulated two sampling strategies (random and regular) across different sample sizes (25, 50, 100 sampling points) with either the ferry route network or the Mediterranean as a sampling frame to compare differences between biodiversity detected by a restricted sampling frame versus unconstrained sampling (Figure 1). Random sampling allocates sampling points anywhere within the sampling frame, whilst regular sampling places sampling points at uniform intervals but introduces randomness with a varied starting point. We explored different combinations of ferry routes, referred to as “ferry subnetworks,” to consider the importance of environmental and species data coverage by the ferry routes. We simulated each sampling strategy combination 1000 times to calculate the mean number of species sampled, and the mean number of occurrences per species in the simulations. All sampling simulations were carried out using the spsample() function from the sp R package to allocate sampling point locations according to the defined sampling frame, strategy, and sampling size (Bivand et al., 2008).

For each sampling strategy simulation, species occurrences were extracted from the “perfect knowledge” SSDM to regenerate new SSDMs from the simulated sampling data, referred to as “sampling SSDMs.” We created these SSDMs as before, except that species were not spatially thinned prior to modeling and species with >20 occurrences were retained. We chose this threshold to evaluate the effect of small sample sizes on model prediction accuracy. To compare species richness across the Mediterranean and ferry route network as sampling frames, 40 replicate SSDMs were built for each combination of the sampling frame, size, and strategy, using 40 different sampling simulations. We assessed the correlation of species richness between the “perfect knowledge” SSDM and the sampling SSDMs based on Pearson correlation coefficient to evaluate the effectiveness of different sampling strategies. A three‐way analysis of variance (ANOVA) was performed to evaluate the effects of sampling strategy, frame, and number of sampling points on the correlation coefficient.

### 2.3 Ferry route subnetworks

We built different ferry subnetworks to evaluate how different coverage of environmental variability and community composition affected the predictive capacity of ferry routes as a sampling frame. The environmental predictors were collapsed into a single index of environmental variability using principal component analysis to quantify the main gradients of environmental variability in the study area (Appendix A; Methods A1). The first four principal components explained >80% of the variability in the environmental predictors. Therefore, we collapsed these principal components by summing the site scores of each principal component weighted according to its contribution (Long & Fisher, 2006; Maina et al., 2008). The resulting environmental variability map was normalized between zero and one, where zero and one represent the most different environments. We quantified climatic bias for different ferry subnetworks by comparing the difference in density functions between environmental variability over the whole study area and those covered by the ferry routes. We split the density functions into five equal bins of 0.2 to calculate the climatic bias index. We define our climatic bias index as the sum of the differences in density functions of environmental variability. Salerno‐Ashdod was the only ferry route that covered the eastern basin and environmental variability between 0 and 0.2. Venice‐Patras was the only ferry route encompassing the Adriatic Sea and environmental variability 0.6–1. These two ferry routes were therefore used to create the environmental subnetwork as they covered all environmental variability in the study area (Figure 2d).

**FIGURE A1:**
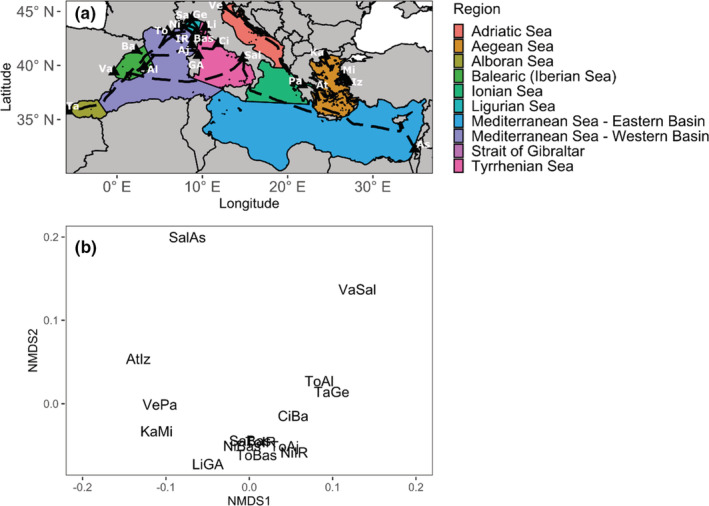
(a) Map of the Mediterranean Sea showing regions covered by different ferry routes. (b) Nonmetric multidimensional scaling plot based on Bray‐Curtis dissimilarity matrix for species composition between different ferry routes.

We also considered how community composition differed between the ferry routes. For each ferry route, species occurrences were extracted from each grid cell of the “perfect knowledge” SSDM that overlapped with the ferry route. The number of grid cells that a species occurred in per route was treated as an abundance estimate. We applied a Hellinger transformation to the resulting species abundance × ferry route matrix to dampen the inflated abundances from longer ferry routes (Legendre & Gallagher, 2001). This transformed matrix was then used to create a Bray–Curtis dissimilarity matrix and differences in species composition between ferry subnetworks were quantified by Nonmetric Multidimensional Scaling (NMDS). The NMDS analysis confirmed, as expected, that ferry routes closer together had more similar species composition, with the main cluster formed from routes in the north‐western basin (Appendix A; Figure A1). This cluster was used to create a deliberately biased ferry route subnetwork (Figure 2b). We also used the NMDS analysis to reduce the number of ferry routes from the original ferry route network by randomly selecting one ferry route from each cluster on the NMDS plot to create a subnetwork representing community composition. This reduced the number of ferry routes in the original network from 15 to 9 (Figure 2c).

We also produced ferry subnetworks with differing numbers of ferry routes, including 2, 4, 6, 8, 10, and 12 ferry routes by randomly selecting routes from the original ferry route network to evaluate the importance of the number of ferry routes. We built 10 sampling SSDMs using 50 regular sampling points per ferry route subset and compared with the “perfect knowledge” SSDM with Pearson correlation coefficient. We assessed the difference between biased, community, and environmental subnetworks, and the difference between subnetworks with differing numbers of ferry routes with one‐way ANOVAs. We performed post‐hoc pairwise comparisons with the Tukey's test.

### 2.4 Taxonomic biases in data collection

The sampling SSDMs were constructed with occurrence data from every sampling point that overlapped with the species distribution. Realistically, no methods for collecting biodiversity data have perfect rates of detectability, so understanding how imperfect detection affects predictions of biodiversity patterns or gradients in biodiversity is important. All biodiversity monitoring techniques, including eDNA metabarcoding, suffer from taxonomic biases (Balint et al., 2018). However, it is unclear how such uncertainty can in turn bias SSDM predictions. To quantify the effect of taxonomic bias, we either removed taxa (Chondrichthyes or Mammalia) or a random subset of species before individual species distribution models were stacked. The random species subset removed the same number of species as the equivalent taxonomically biased model. The models were then compared with the “perfect knowledge” SSDM using Pearson correlation coefficient. We analyzed the effect of removing specific taxa with a three‐way ANOVA and post‐hoc pairwise comparisons with the Tukey's test.

## 3 RESULTS

### 3.1 Stacked species distribution model

**FIGURE 3:**
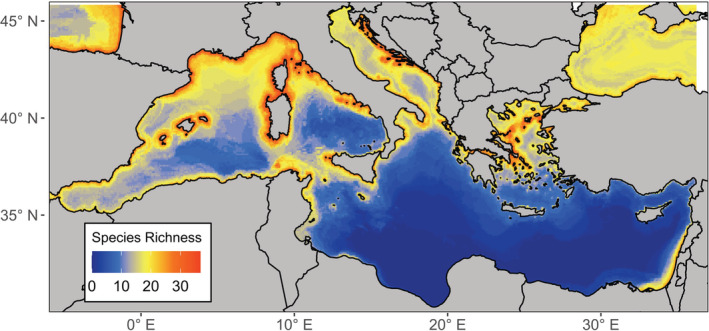
Original binary stacked species distribution model of 43 marine predators in the Mediterranean using occurrence data obtained from online repositories.

The SSDM of marine predators in the Mediterranean revealed two main gradients in species richness (Figure 3). There was higher species richness in the north‐western basin than in the south‐eastern basin, and higher species richness nearer to shore. The environmental variable with the greatest influence on model predictions was mean sea surface temperature, whilst the variable with the least influence was bathymetric slope (Appendix A; Table A2). The remaining variables, mean bathymetry, mean chlorophyll concentration, mean temperature range, and distance from shore, contributed equally to model predictions. The model tended to overpredict species richness although the extent varied greatly (species richness error mean = 19.06 ± 7.23 SD). The proportion of presences that were correctly predicted (sensitivity = 0.98 ± 0.12 SD) was much higher than the proportion of absences correctly predicted (specificity = 0.54 ± 0.17 SD) (Appendix A; Table A3).

### 3.2 Comparison of ferry route sampling frame to whole Mediterranean

**FIGURE 4:**
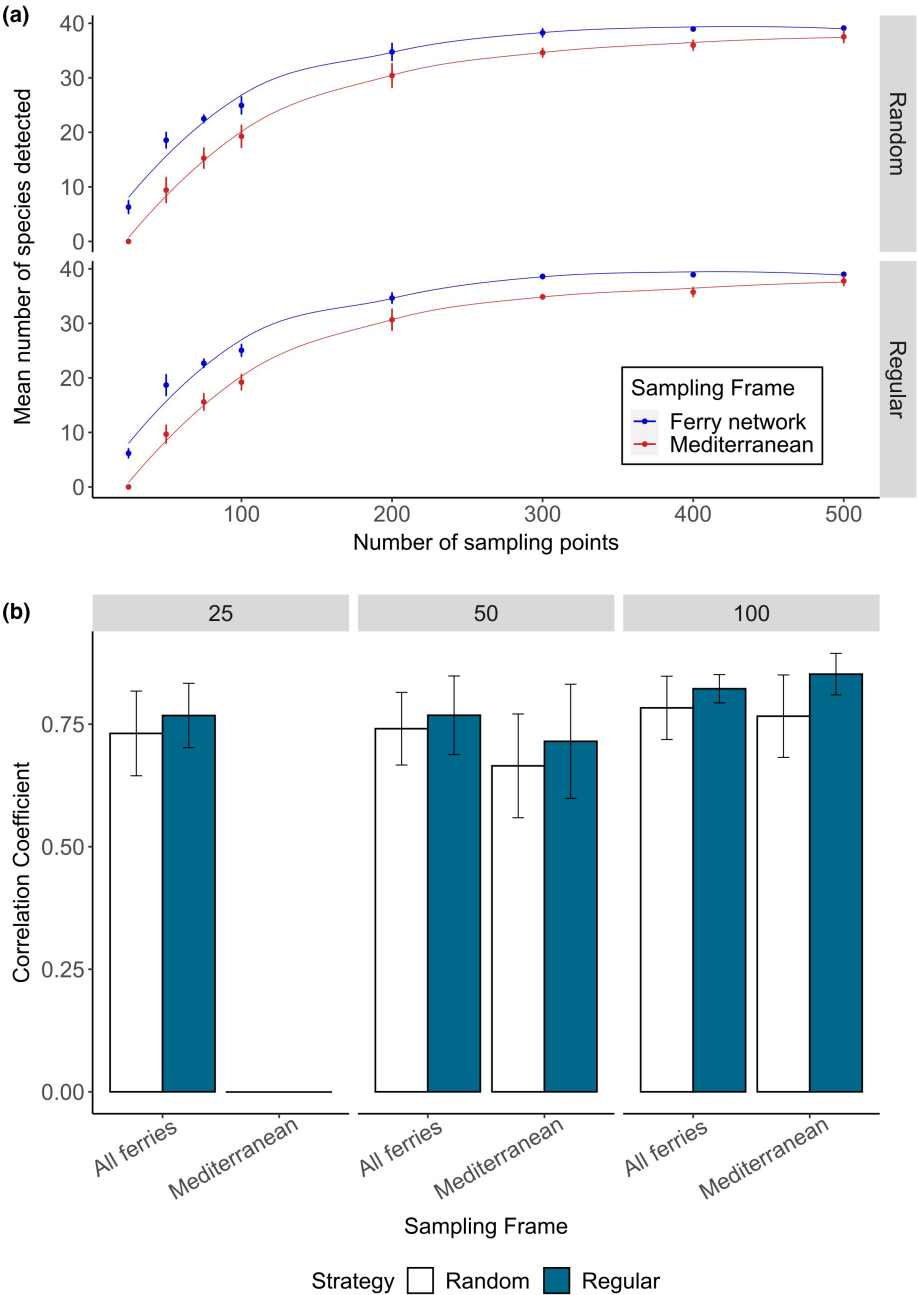
(a) The mean number of species detected, with standard deviation bars, across the different number of sampling points using either the ferry network or Mediterranean as a sampling frame and either a random or regular sampling strategy. (b) Mean Pearson correlation coefficient between the original SSDM and sampling SSDMs for 40 replicate simulations across the ferry network and Mediterranean for two sampling strategies (random and regular) across three different sample sizes (25, 50, and 100 sampling points). There was not enough occurrence data with 25 sampling points and the Mediterranean as a sampling frame to remake SSDMs.

The number of species with enough occurrences for modeling (>20) was consistently higher for samples collected along the ferry route network compared with unconstrained sampling across the Mediterranean Sea (Figure 4a). For the smallest number of sampling points (25), only the ferry routes could detect any species with enough occurrence points for modeling (random = 6.3 ± 1.27 SD, regular = 6.17 ± 0.91 SD). With 50 sampling points, the ferry routes (random = 18.56 ± 1.53, regular = 18.69 ± 1.99 SD) detected double the amount of species compared with the Mediterranean (random = 9.42 ± 2.37, regular = 9.67 ± 1.74). The sampling strategy, random vs regular, had no effect on the number of species detected in both the ferry route and whole Mediterranean simulated sampling. The number of species detected increased quickly at small sample sizes but asymptotes between 200 and 500 sampling points where only five new species were detected using the ferry route network, and seven species using the Mediterranean.

Sampling SSDMs created from 100 regular sampling points across the Mediterranean were most correlated to the “perfect knowledge” SSDM (85.2% ± 4 SD) (Figure 4b). Sampling SSDMs produced from 100 sampling points collected regularly (82.2% ± 3 SD) or randomly (78.3% ± 6 SD) across the ferry route network also produced SSDMs highly correlated with the “perfect knowledge” SSDM. Sample size and sampling strategy had less effect on the predictive capacity of sampling SSDMs produced with the ferry route network compared with the Mediterranean Sea. Sampling SSDMs created with either 25 or 50 sampling points along the ferry routes correlated more with the “perfect knowledge” SSDM compared with 50 sampling points across the Mediterranean Sea regardless of sampling strategy (*F*_(1,373)_ = 15.8, *p* < .001). Sampling SSDMs created from randomly allocated sampling points correlated less with the “perfect knowledge” SSDM compared with sampling SSDMs with regularly spaced sampling points. The difference in predictive capacity between the two sampling strategies was greater for samples collected using the Mediterranean instead of the ferry route network (*F*_(1,373)_ = 3.91, *p* = .05) (Table 1).

**TABLE 1:** Three‐way ANOVA table to evaluate the impact of the sampling strategy, sampling frame, and number of sampling points on correlation coefficients between the sampling SSDMs and the “perfect knowledge” SSDM.

Factor	Df	Sum Sq	Mean Sq	*F* value	*p* value
Strategy	1	0.4564	0.45651	37.4637	<.001*
Size	2	1.1520	0.57601	47.2701	<.001*
Sampling frame	1	0.0960	0.09603	7.8804	.005*
Strategy:Size	2	0.0471	0.02355	1.9324	.15
Strategy:Sampling frame	1	0.0477	0.04770	3.9147	.049*
Size:Sampling frame	1	0.1925	0.19249	15.7964	<.001*
Strategy:Size:Sampling frame	1	0.0097	0.00966	0.7927	.37

### 3.3 Ferry route subnetworks

**FIGURE 5:**
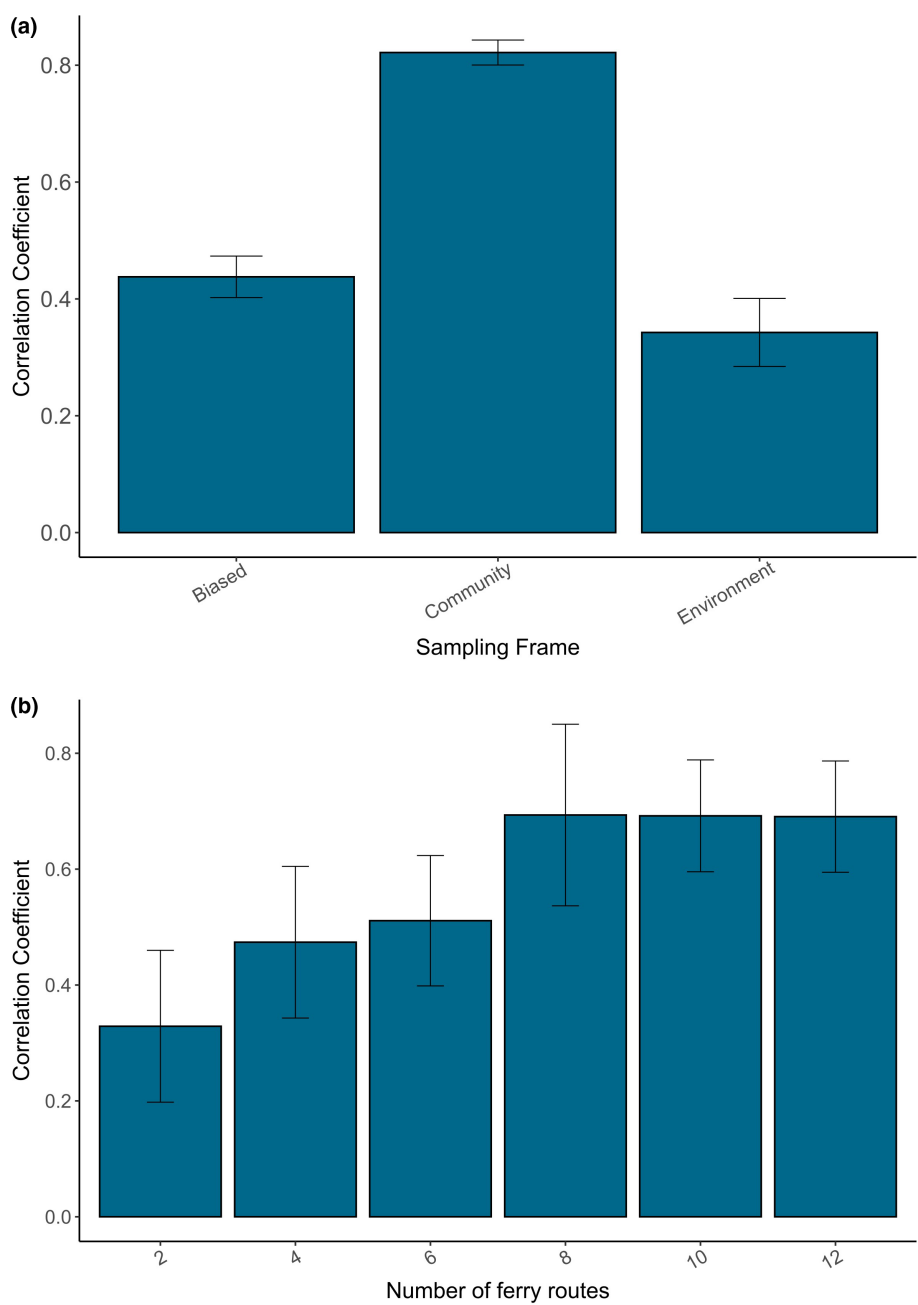
(a) Mean Pearson correlation coefficient between the original SSDM and sampling SSDMs for 10 replicate simulations across ferry route subnetworks using 50 regular sampling points. (b) Mean Pearson correlation coefficient between original SSDM and sampling SSDMs for 10 replicate simulations across subnetworks with differing numbers of ferry routes.

Different ferry subnetworks varied in their ability to accurately capture community composition in the “perfection knowledge” SSDM (*F*_(1, 26)_ = 342.96, *p* < .001) (Figure 5a; Appendix A; Table A4). The community subnetwork was able to predict the original community composition ~40% better than either the biased or environment subnetworks (Tukey's, *p* < .05). The community subnetwork also had a similar climatic bias index to the network with all ferry routes included (Appendix A; Table A5). The environment subnetwork predicted community composition (34.3% ±0.6) ~9% worse than the deliberately biased subnetwork (43.8% ±0.4) (Tukey's, *p* < .05). The deliberately biased sampling strategy had the highest climatic bias index, whilst the environment subnetwork performed similarly to the original ferry network (Appendix A; Table A5). The number of ferry routes included in a network affected its predictive capacity (*F*_(1, 54)_ = 15.286, *p* < .001), with correlation to the “perfection knowledge” SSDM increasing from 32.8% ±13 for networks with two ferry routes, to 69.3% ±16 with 8 ferry routes (Tukey's, *p* < .001) (Figure 5b). Increasing beyond eight routes does not improve the predictive capacity of the sampling frame but reduces the variability related to which ferry routes are selected in the subnetwork (Tukey's, *p* > .05).

### 3.4 Taxonomic biases in data collection

**FIGURE 6:**
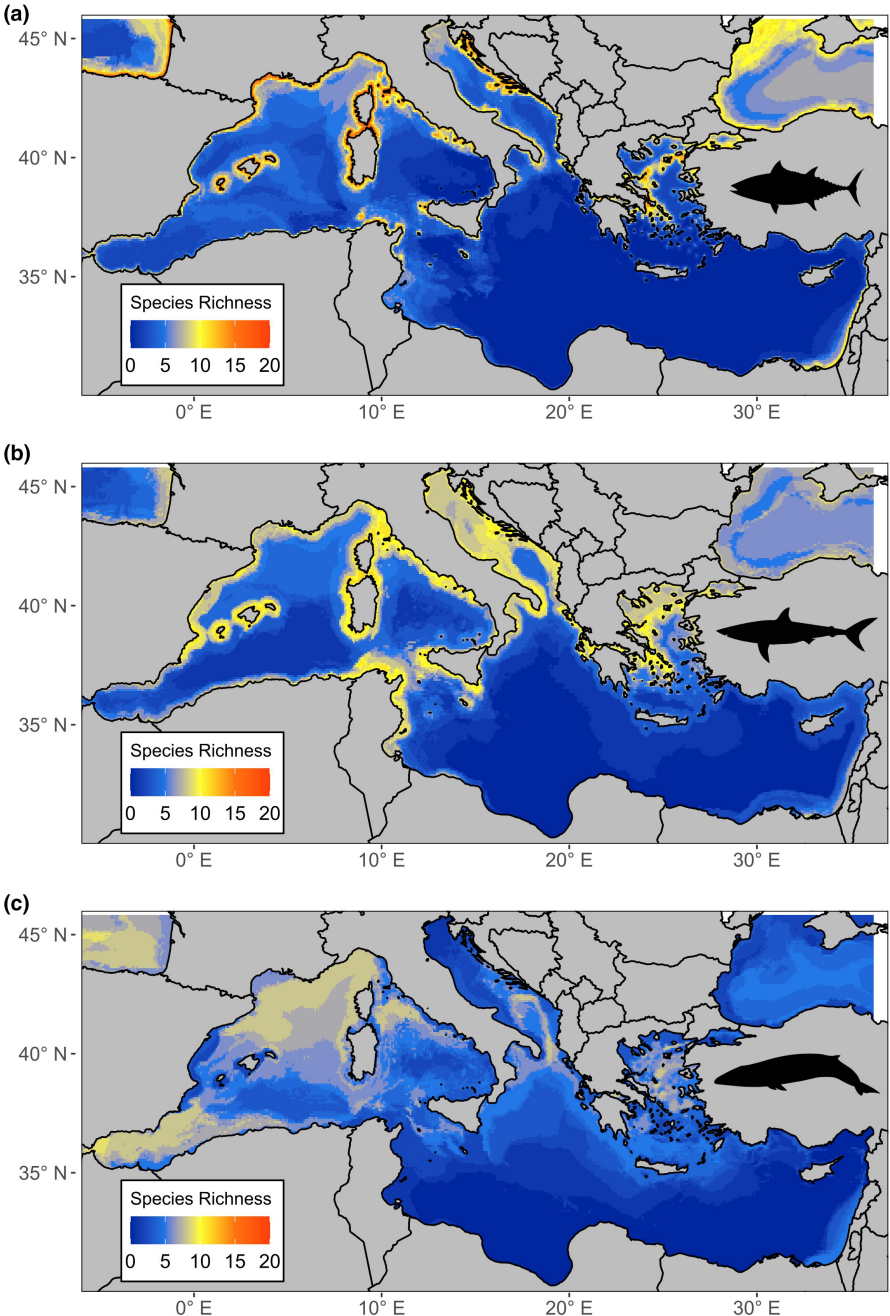
Stacked species distribution models for Class (a) Actinopterygii, (b) Chondrichthyes, (c) Mammalia.

**FIGURE 7:**
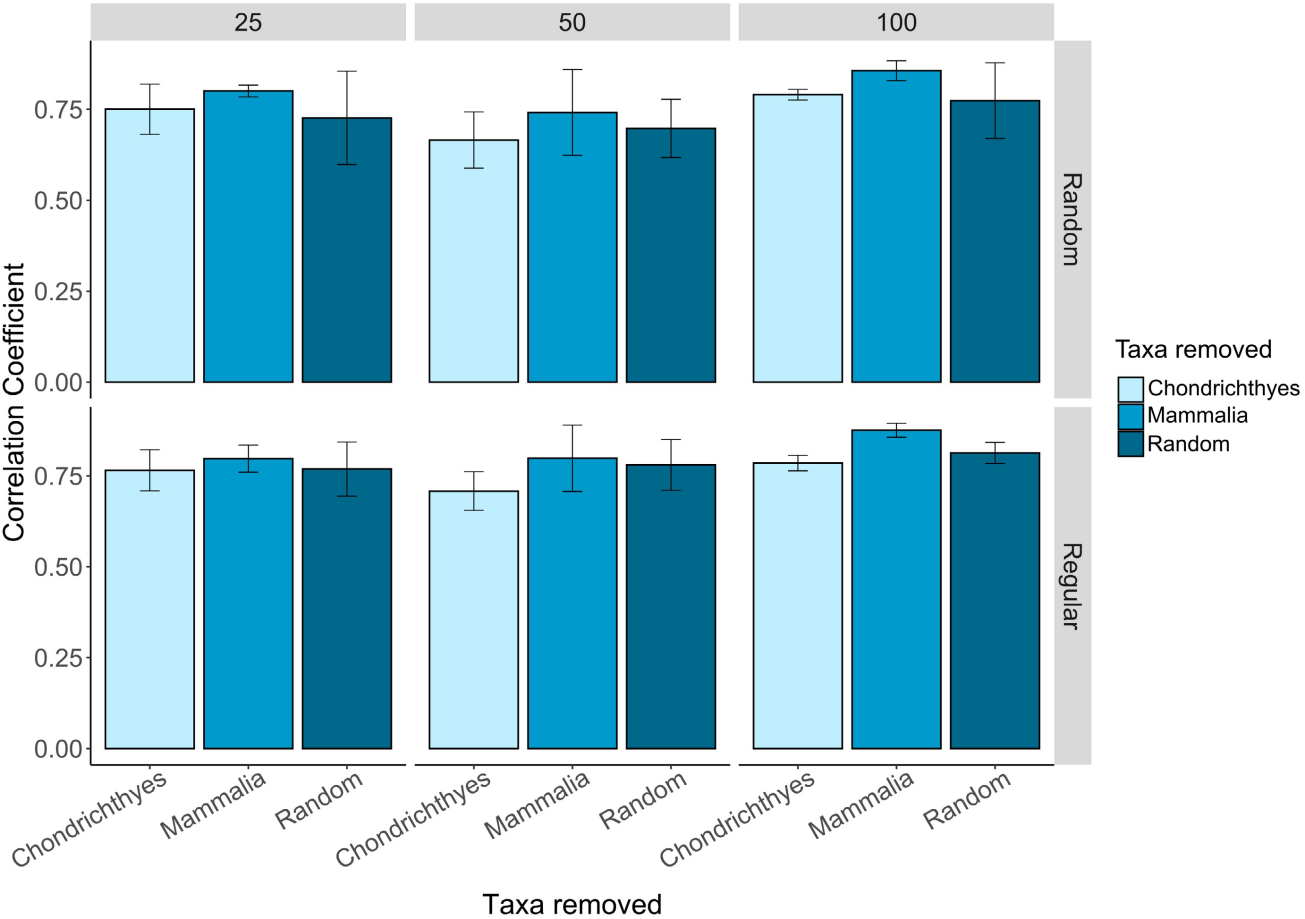
Mean Pearson correlation coefficient between the original SSDM and sampling SSDMs with different taxonomic biases for samples collected using the ferry network sampling frame.

When species in the same class were stacked together and compared with the “perfect knowledge” SSDM, Actinopterygii (91.9%) and Chondrichthyes (90.9%) both had similar species richness patterns to the “perfect knowledge” SSDM (Figure 6). The Mammalia‐only SSDM (67.96%) showed a weaker correlation with the “perfect knowledge” SSDM. Sampling SSDMs with different taxa removed affected the predicted community composition (*F*_(1,116)_ = 8.72, *p* < .001) (Figure 7). Sampling SSDMs with Mammalia species removed improved the predictive capacity by 10% compared with sampling SSDMs with Chondrichthyes removed, or by 7% compared with a random subset of species removed (Tukey's, *p* < .001). This pattern was consistent across a range of sampling sizes and strategies.

## 4 DISCUSSION

Biased sampling remains a key hurdle to predicting biodiversity patterns (Hughes et al., 2021; Moussy et al., 2022; Tydecks et al., 2018). We evaluated the feasibility of using biased sampling frames (in this case commercial vessels) as sampling platforms for collecting species occurrence data for marine species distribution modeling. In this study, we test ferry routes that could offer low‐cost access to vessels (compared with dedicated research cruises) for hard‐to‐reach pelagic regions but introduce biases because spatial sampling is restricted to the routes covered. We found that the inherent bias associated with restricted sampling frames did not lead to a loss in predictive capacity. In fact, for our case study, sampling simulations with ferry routes recovered species richness gradients more accurately than unconstrained sampling at small (25 sampling points) and medium (50 sampling points) sample sizes as a result of ferry routes constraining sampling to areas with higher biodiversity. This result further highlights the cost‐effectiveness of ferry routes as sampling platforms and demonstrates that high‐quality biodiversity data can be recovered from restricted sampling frames. Implementing this workflow to design surveys across the global shipping network, including from other vessel types (e.g., container ships), could vastly expand our knowledge of marine biodiversity in inaccessible areas, and is especially applicable for expanding the spatiotemporal scale of emerging techniques, such as automated environmental DNA sampling (Valsecchi et al., 2021).

### 4.1 Marine predator SSDM

**FIGURE A2:**
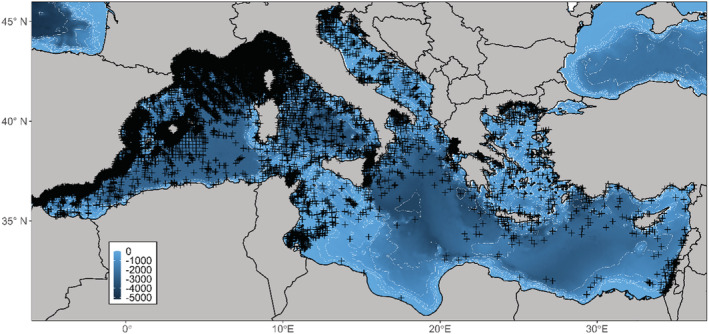
Bathymetric map of the Mediterranean Sea showing raw occurrence data from the online data repositories GBIF, OBIS and EurOBIS as well as the ACCOBAMS and Medlem datasets for cetaceans and elasmobranchs respectively.

The SSDM shows that predator species richness is much higher in the north‐western basin (Figure 3). This result is unsurprising due to the Strait of Gibraltar linking the western basin to the Atlantic Ocean allowing the migration of predators into the Mediterranean (Coll et al., 2010). Critical habitat, including breeding and foraging grounds, for marine predators has been recognized in the north‐western basin through Ecologically or Biologically Significant Areas (EBSAs), and the implementation of the Pelagos Sanctuary for Marine Mammals (Notarbartolo di Sciara et al., 2008; UNEP/CBD/EBSA/WS/2014/3/4, 2014). However, there was also a greater density of occurrence points used to create the “perfect knowledge” SSDM in the north‐western region compared with offshore and in the southern basin (Appendix A; Figure A2). This sampling bias is driven by greater economic resources in northern basin countries, which benefit from European Union (EU) funding for survey and conservation initiatives (Amengual & Alvarez‐Berastegui, 2018; Coll et al., 2010). The binary SSDM tended to overpredict species richness, as has been previously reported (Pottier et al., 2013). Combining SSDMs with macroecological constraints may reduce overprediction by accounting for biotic interactions (d'Amen et al., 2015; Guisan & Rahbek, 2011). However, SSDMs can provide similar predictions to macroecological models or joint species distribution models when using a probabilistic stacking approach (Calabrese et al., 2014; Zurell et al., 2020). Despite its limitations, we chose to use a binary stacking procedure as we required presence data to re‐run species distribution models from the simulated sampling strategies and the model represents realistic community patterns as a base for sampling simulations.

### 4.2 Comparison of ferry route sampling frame to whole Mediterranean

Our selected 15 operational ferry routes are assumed to be representative of the spatial extent of the Mediterranean‐wide ferry network (Figure 2a). Using this ferry route network as a sampling frame achieved species distribution models that predicted the known community from the “perfect knowledge” SSDM or as well as or better than samples collected across the whole Mediterranean. Ideally, occurrence data for species distribution modeling would represent a random sample from the population of interest across the entire study area (Araujo & Guisan, 2006). However, geographically biased sampling strategies, i.e., samples only collected close to road networks, can still produce accurate models as long as the environmental predictors are not also biased, as is the case with the ferry route network (Kadmon et al., 2004; Tessarolo et al., 2014). Here, we demonstrate that with smaller sample sizes, samples collected from the biased sampling frame produced more accurate models than samples collected from across the whole Mediterranean Sea (Figure 4b). It is more feasible to routinely collect samples on board ferries than to implement dedicated research surveys over large spatial scales comparable to the Mediterranean Sea. Therefore, we show that routine sampling on ferries can serve as an important approach to conduct representative biodiversity sampling.

Fewer samples are required to produce models with similar accuracy from ferry routes compared with the whole Mediterranean, but smaller sample sizes result in less species being detected. For the ferry route network, there is no cost benefit to doubling the sample size as this does not improve the SSDM community composition prediction (Figure 4). However, the SSDM made with 25 sampling points only detected between 5–8 species (11–18%) whereas SSDMs with 50 sampling points detected 16–21 (37–48%) species, and SSDMs with 100 sampling points detected 19–26 species (44–60%) (Figure 4). If the aim of the study is to look at patterns in species richness, such as gradients in diversity, then a small sample size is adequate. However, if individual species distributions, or the detection of rare species is also important, then larger sample sizes will be required. These sample sizes are based on 100% detection rates of the species when they are present, which is unrealistic for any sampling method. However, we expect that the patterns observed between sample sizes and sampling frames should hold true as long as the detection probabilities are constant across sampling frames. Sampling SSDMs from the ferry networks were less affected by the sampling strategy than sampling SSDMs from the whole Mediterranean, where random sampling consistently produced more poorly performing SSDMs. By limiting the available sampling frame to such an extent, this potentially reduces the impact of the sampling strategy and prevents random sampling from forming clusters that do not cover the study area's environmental variability (Zhang et al., 2020). These results suggest that ferries, or other commercial shipping routes, represent a promising sampling platform to alleviate constraints on access to pelagic environments that currently limit marine biodiversity surveys.

### 4.3 Differences between ferry routes and subnetworks

Environmental variability and species composition were compared between individual ferry routes to understand which ferry routes were important when building a subnetwork. The routes between Salerno‐Ashdod and Venice‐Patras were the only two routes that covered the extremities of environmental variability and so were required in any ferry subnetwork to achieve full coverage of the environmental parameter space. Previous research suggests that sampling frames can be geographically biased as long as the full range of environmental variability in the whole sampling area is covered (Kadmon et al., 2004; Tessarolo et al., 2014). However, our results demonstrate that the environment subnetwork was not able to accurately predict community composition despite covering environmental variability, and in fact performed similarly to the deliberately biased subnetwork (Figure 5a). This highlights that considering environmental variability alone may not reduce the biases associated with restricted sampling frames. The NMDS analysis showed that the routes covering Salerno‐Ashdod and Venice‐Patras do not cluster with any other routes suggesting that different species compositions occur on these routes (Appendix A; Figure A1). Meanwhile, the community subset, which covered both community composition and environmental variability, predicted species richness in the “perfection knowledge” SSDM most accurately. This result highlights that community composition and environmental variability must be considered when selecting ferry routes to be representative sampling frames.

The original ferry route network had a high density of shipping routes in the northwest Mediterranean, coinciding with the region with most biodiversity data available, which we expected to bias the predictive capacity of the sampling SSDMs using this network (Figure 2). However, the community subnetwork, with six routes removed from the northwest basin, was still able to accurately predict community composition suggesting this was not a driving factor in the effectiveness of the ferry routes as a sampling frame (Figure 5). A limitation of using existing community composition knowledge to select ferry routes for sampling is that it requires reliable occurrence data to model a “perfect knowledge” SSDM. Here, the NMDS analysis shows that community composition along the ferry routes is related to the geographical location of the routes, with routes closer together having more similar community composition (Appendix A; Figure A1). We also demonstrated that increasing the number of routes within the network, and having fewer sampling points along more routes, will lead to improved predictions of community composition. Therefore, we recommend implementing a large number of ferry routes, at least 8, that cover as many different regions of a study area as possible if pre‐existing occurrence data are unreliable or limited.

### 4.4 Random and systematic biases in data collection

Reports identifying taxonomic biases in biodiversity surveys are pervasive in the literature, but little is known about how taxonomic biases can affect downstream analyses such as species distribution modeling or spatial planning (Di Marco et al., 2017; Donaldson et al., 2016; Troudet et al., 2017). Instead, efforts to reduce bias in species distribution models have largely been directed at spatial and temporal biases in data collection (Beck et al., 2014; Inman et al., 2021; Kramer‐Schadt et al., 2013). We demonstrate that different taxa have varying species richness gradients, thus removing different taxonomic groups affected which species richness gradients were revealed. The classes Actinopterygii (fishes) and Chondrichthyes (sharks and rays) both showed the highest species richness closest to shore whereas marine mammals were more prevalent offshore. This is unsurprising as Actinopterygii and Chondrichthyes are more closely related, and are largely ectothermic so more constrained by temperature requirements than marine mammals (Grady et al., 2019; Losos, 2008). However, this may have been exaggerated by the greater availability of marine mammal data offshore from visual ferry surveys compared with Actinopterygii and Chondrichthyes data, which is largely collected by coastal fisheries (Aïssi et al., 2015; Mancusi et al., 2020). Models with marine mammals removed were more correlated to the “perfect knowledge” SSDM as a result of more species belonging to the Actinopterygii and Chondrichthyes classes than marine mammals (Figure 7). This highlights that the proportion of species representing each class has an important influence on the overall species richness gradients captured. If biases lead to certain taxonomic groups being underrepresented, then it is unlikely that their species richness gradients would be adequately captured, unless they follow similar distributions to another taxa. To utilize novel methods for biodiversity data collection most effectively, it is important to understand the effect taxonomic bias can have, and how new methods can best reduce current biases.

## 5 CONCLUSION/FUTURE RESEARCH

Our study demonstrates that high‐quality biodiversity data can be collected from biased sampling frames, providing they cover wide areas and diversified habitats. Utilizing these biased sampling frames, such as ferries, allows data collection from challenging and remote areas, which are often inaccessible to researchers due to logistical and financial constraints. This is particularly relevant for upscaling sampling for emerging biodiversity monitoring techniques, such as automated eDNA sampling, to reduce current spatial, temporal, and taxonomic biases (Pawlowski et al., 2020). This study focused on the ferry routes in the Mediterranean to carry out simulated sampling strategies, but sampling from ferry routes, as well as other commercial vessel types, could be carried out across the global shipping network. The efficiency of ferry routes as sampling platforms will depend on the concentration of ferry routes in the study area or region of interest. Global cargo routes are largely concentrated in the North Atlantic, North Pacific, and Indian Oceans, linking Europe, North America, East, and Southeast Asia. High traffic routes crossing the South Atlantic and South Pacific also connect with Southern Africa, South America, and Australasia. These represent key areas where commercial vessels could contribute to closing gaps in biodiversity data (Wang & Wang, 2011). These areas also coincide with those most affected by human impacts emphasizing the need for regular monitoring to understand the effects on biodiversity (Halpern et al., 2008; Pirotta et al., 2019). The workflow presented here can be used as a template to evaluate the efficiency of a shipping route network in a study area of interest before undertaking sampling. This study focused on the impact of sampling strategies on species distribution models, which are frequently used as conservation features in marine spatial planning to designate protected areas. Therefore, our findings confirm that biased sampling, if designed adequately, can provide a useful data basis for marine species and the management of marine environments.

## AUTHOR CONTRIBUTIONS

**Elizabeth Boyse:** Data curation (lead); formal analysis (lead); methodology (equal); visualization (lead); writing – original draft (lead); writing – review and editing (equal). **Maria Beger:** Conceptualization (equal); methodology (equal); supervision (equal); writing – review and editing (equal). **Elena Valsecchi:** Supervision (supporting); writing – review and editing (supporting). **Simon J Goodman:** Conceptualization (equal); methodology (equal); supervision (equal); writing – review and editing (equal).

### OPEN RESEARCH BADGES

This article has earned an Open Data badge for making publicly available the digitally‐shareable data necessary to reproduce the reported results. The data is available at [https://doi.org/10.5061/dryad.280gb5ms5].

## Data Availability

Individual marine predator species distribution models and the binary stacked species distribution model are available at https://doi.org/10.5061/dryad.280gb5ms5. Code used to execute this study is available at https://github.com/eboyse/Ferries_simulated_sampling_strategies.

## APPENDIX A

**TABLE A1:** List of 43 marine predator species that had >40 occurrence points from combined data from online repositories, GBIF, OBIS, and EurOBIS, Accobams, and the Medlem database.

Species	Common name	Class	Total length (cm)	Trophic level	Number of occurrences
*Conger conger*	European Conger	Actinopterygii	573.3	4.3	649
*Dentex dentex*	Common dentex	Actinopterygii	100	4.5	372
*Echelus myrus*	Painted eel	Actinopterygii	100	4.3	45
*Epinephelus aeneus*	White grouper	Actinopterygii	120	4	49
*Epinephelus marginatus*	Dusky grouper	Actinopterygii	150	4.4	969
*Fistularia commersonii*	Bluespotted cornetfish	Actinopterygii	160	4.3	63
*Lophius piscatorius*	Angler	Actinopterygii	200 (SL)	4.5	602
*Merluccius merluccius*	European hake	Actinopterygii	140	4.4	1179
*Mola mola*	Ocean sunfish	Actinopterygii	333	3.3	3530
*Molva dypterygia*	Blue ling	Actinopterygii	155	4.5	119
*Muraena helena*	Mediterranean moray	Actinopterygii	150	4.2	1061
*Ophisurus serpens*	Serpent eel	Actinopterygii	250	4.1	47
*Pomatomus saltatrix*	Bluefish	Actinopterygii	130	4.5	82
*Seriola dumerili*	Greater amberjack	Actinopterygii	190	4.5	118
*Sphyraena sphyraena*	European barracuda	Actinopterygii	165	4	159
*Sphyraena viridensis*	Yellowmouth barracuda	Actinopterygii	128 (FL)	4.3	54
*Thunnus alalunga*	Albacore	Actinopterygii	140 (FL)	4.3	270
*Thunnus thynnus*	Bluefin tuna	Actinopterygii	458	4.5	177
*Xiphias gladius*	Swordfish	Actinopterygii	455	4.5	48
*Zu cristatus*	Scalloped ribbonfish	Actinopterygii	118 (SL)	4.5	204
*Alopias vulpinas*	Common thresher	Chondrichthyes	573.3	4.5	114
*Carcharhinus longimanus*	Oceanic whitetip shark	Chondrichthyes	400	4.2	77
*Cetorhinus maximus**	Basking shark	Chondrichthyes	1520	3.2	142
*Dasyatis pastinaca**	Common stingray	Chondrichthyes	64 (WD)	4.1	163
*Echinorhinus brucus*	Bramble shark	Chondrichthyes	310	4.4	41
*Hexanchus griseus*	Bluntnose sixgill shark	Chondrichthyes	482	4.5	140
*Isurus oxyrinchus*	Short‐fin mako shark	Chondrichthyes	445	4.5	81
*Mobula mobular**	Giant devil ray	Chondrichthyes	520 (WD)	3.7	874
*Myliobatis aquila**	Common eagle ray	Chondrichthyes	183 (WD)	3.6	119
*Prionace glauca*	Blue shark	Chondrichthyes	400	4.4	324
*Raja clavata**	Thornback ray	Chondrichthyes	139	3.8	431
*Squalus acanthias**	Spiny dogfish	Chondrichthyes	95	4.4	78
*Torpedo marmorata*	Marbled electric ray	Chondrichthyes	100	4.5	116
*Balaenoptera physalus*	Fin whale	Mammalia	2700	3.2–4.3	1245
*Delphinus delphis*	Short‐beaked common dolphin	Mammalia	260	4.5	1693
*Globicephala melas*	Long‐finned pilot whale	Mammalia	670	4.5	1147
*Grampus griseus*	Risso's dolphin	Mammalia	380	4.36–4.54	410
*Orcinus orca*	Killer whale	Mammalia	980	4.5–4.6	115
*Physeter macrocephalus*	Sperm whale	Mammalia	2400	4.5–4.7	2307
*Stenella coeruleoalba*	Striped dolphin	Mammalia	260	4.5	7822
*Tursiops truncatus*	Bottlenose dolphin	Mammalia	380	4.5	3991
*Ziphius cavirostris*	Cuvier's beaked whale	Mammalia	750	4.5	113
*Caretta caretta**	Loggerhead turtle	Reptilia	125 (CL)	3.5–3.6	1557

*Note*: Total length and tropic level as reported by FishBase (Actinopterygii and Chondrichthyes) or SeaLifeBase (Mammalia and Reptilia). Marine predators defined as having total length ≥ 1 m and trophic level ≥ 4. Species that do not meet these criteria but were retained are denoted with an Asterix.

Ray species *D. pastinaca* and *Torpedo torpedo* were retained due to having trophic levels greater than 4. Total length is not reported for these species so retained despite their width being less than 1 metre.

*R. clavata* and *S. acanthias* were retained as they were so close to threshold.

*Mola mola, Cethorhinus maximus, Mobular mobular, Myliobatis aquila*, and *Caretta caretta* were retained as they are classed as marine megafauna species, which can exert top‐down effects on ecosystems, similar to that of predators (Pimiento et al., 2020).

Pimiento, C., Leprieur, F., Silvestro, D., Lefcheck, J., Albouy, C., Rasher, D., Davis, M., Svenning, J.‐C., and Griffin, J. 2020. Functional diversity of marine megafauna in the Anthropocene. *Science Advances*. **6**(16), peaay7650.

### A.1. Methods

Principal component analysis (PCA) was used to create an environmental variability map. Initially, PCA was conducted on a correlation matrix of standardized environmental predictors used to create the SSDM using the prcomp() function in the “stats” R package. The first four principal components were retained for downstream analysis as they explained >80% of the variability in the environmental predictors. Site scores, i.e., weighted linear combinations of the environmental predictors, were used to produce surface maps of each of the first four principal components to visualize the main gradients of environmental variability in the study area. The principal components were collapsed into one surface map of environmental variability by summing the site scores of each principal component weighted according to its contribution as following the equation:

$$\mathrm{EV}=\left(0.3665^{*}\text{PC1}\right)+\mathrm{inv}\left(0.2435^{*}\text{PC2}\right)+\left(0.164^{*}\text{PC3}\right)+\left(0.1121^{*}\text{PC4}\right)$$

Mean bathymetry made an important contribution to PC1 and PC2 (factor loading >0.4) but a high score in PC1 related to shallow bathymetry while a high score in PC2 related to greater depths. Therefore, PC2 was inverted otherwise the site scores offset each other and the variability was lost. The final “environmental variability map” is unitless and shows the main gradients in environmental variability in the study area.

The first principal component explained 36.65% of the variability in the environmental predictors where the highest values correspond to shallow bathymetry and low sea surface temperatures but high values of chlorophyll concentration and sea surface temperature range. The second principal component explained 24.35% of the variability and represents variability related to distance from shore, where bathymetry is deepest further from shore. The third principal component explained 16.4% of the variation where larger values correlated to the largest bathymetric slope. The fourth principal component explained 11.21% of the variation with the northern Adriatic clearly being the most different area due to having a high chlorophyll concentration and lower sea surface temperature.

The weighted overlay of the principal components shows the overall trends in environmental variability where the values are unitless, but the larger the range between values represents areas with the most different environmental conditions. There are two main trends in environmental variability, (1) a difference between the north‐western and south‐eastern basins, (2) a gradient with distance from shore. The northern tip of the Adriatic Sea is clearly most different from the rest of the Mediterranean.

Principal component respective contribution ratios.

**Table:**

	PC1	PC2	PC3	PC4	PC5	PC6
Eigenvalue	1.4828	1.2088	0.992	0.8203	0.65213	0.50760
Contribution ratio (%)	0.3665	0.2435	0.164	0.1121	0.07088	0.04294
Cumulative contribution (%)	0.3665	0.6100	0.774	0.8862	0.95706	1.0000

Eigenvectors. Factor loadings >0.4 are highlighted in bold.

**Table:**

Variable (units)	PC1	PC2	PC3	PC4
Mean bathymetry (m)	**−0.4281**	**0.5227**	−0.2578	0.0677
Mean sea surface temperature (°C)	**−0.4773**	−0.3344	0.3005	0.2529
Mean chlorophyll concentration (mg/m^3^)	**0.4436**	0.1910	−0.0693	**0.8660**
Sea surface temperature range (°C)	**0.4783**	0.3533	−0.1049	**−0.4166**
Bathymetric slope (°)	−0.1490	−0.2761	**−0.9079**	0.0511
Distance to shore (km)	−0.3756	**0.6143**	0.0568	0.0735

Environmental variability map using weighted overlay of the first four principal components.

Principal component scores projected onto the study area.

**TABLE A2:** Pearson correlation coefficient calculated from the difference between a full model and one with each environmental variable omitted in turn for individual species models then averaged across species.

	Mean bathymetry	Mean sea surface temperature	Mean chlorophyll concentration	Mean temperature range	Bathymetric slope	Distance from shore
Mean	14.59	31.76	13.17	16.7	8.31	15.47
SD	6.56	13.74	5.1	5.53	1.96	8.32

**TABLE A3:** Results from six metrics used to evaluate the prediction accuracy of species assemblage predictions in the “perfect knowledge” SSDM.

	Species richness error	Prediction success	Kappa	Specificity	Sensitivity	Jaccard
Mean	19.06	5.98	0.995029	0.544447	0.983425	0.065596
SD	7.229589	1.797052	0.000441	0.172317	0.124641	0.059542

**TABLE A4:** Analysis of variance tables.

One‐way ANOVA to evaluate the impact of different ferry route subnetworks on correlation coefficients.					
Factor	Df	Sum Sq	Mean Sq	*F* value	*p* value
Sampling frame	2	1.20122	0.60061	342.96	<.001*

Tukey's post‐hoc test to evaluate which ferry route subnetworks differed from each other.					
Group 1	Group 2	Estimate	Conf. Low	Conf. High	*p* value
Biased	Community	0.384	0.336	0.432	<.001*
Biased	Environment	−0.0952	−0.142	−0.0487	<.001*
Community	Environment	−0.479	−0.527	−0.431	<.001*

One‐way ANOVA to evaluate the effect of the number of ferry routes in a ferry subnetwork. Dependent variable was square transformed prior to analysis.					
Factor	Df	Sum Sq	Mean Sq	*F* value	*p* value
Number of ferries	5	1.2987	0.259745	15.286	<.001*

Tukey's post‐hoc test to evaluate which ferry subnetworks differed depending on the number of ferry routes.					
Group 1	Group 2	Estimate	Conf. Low	Conf. High	*p* value
2	4	0.116	−0.0558	0.289	ns
2	6	0.149	−0.0233	0.321	ns
2	8	0.379	0.207	0.552	<.001*
2	10	0.364	0.191	0.536	<.001*
2	12	0.362	0.189	0.534	<.001*
4	6	0.0325	−0.140	0.205	ns
4	8	0.263	0.0907	0.435	<.001*
4	10	0.247	0.0750	0.419	<.001*
4	12	0.245	0.0730	0.418	<.001*
6	8	0.230	0.0582	0.403	<.001*
6	10	0.215	0.0425	0.387	<.001*
6	12	0.213	0.0405	0.385	<.001*
8	10	−0.0157	−0.188	0.157	ns
8	12	−0.0177	−0.190	0.155	ns
10	12	−0.00194	−0.174	0.170	ns

Three‐way ANOVA to evaluate the impact of removing specific taxa from stacked species distribution models across sampling strategies (random vs regular) and sampling sizes (25, 50, 100 sampling points). Dependent variable (the correlation coefficient) was square transformed prior to analysis.					
Factor	Df	Sum Sq	Mean Sq	*F* value	*p* value
Strategy	1	0.08385	0.083854	7.9500	<.05*
Size	2	0.26295	0.131475	12.4648	<.001*
Taxa removed	2	0.18388	0.091941	8.7167	<.001*

Tukey's post‐hoc test to look at pairwise differences between taxa removed, sampling strategy, and sampling size.						
Term	Group 1	Group 2	Estimate	Conf. Low	Conf. High	*p* value
Strategy	Random	Regular	0.0522	0.0155	0.0889	<.05*
Size	25	50	−0.0400	−0.0936	0.0136	ns
Size	25	100	0.0715	0.0170	0.126	<.05*
Size	50	100	0.112	0.0580	0.165	<.001*
Taxa removed	Chondrichthyes	Mammalia	0.106	0.0428	0.169	<.001*
Taxa removed	Chondrichthyes	Random	0.0299	−0.0241	0.0840	ns
Taxa removed	Mammalia	Random	−0.0758	−0.130	−0.0217	<.05*

*Note*: * indicates significant variables.

**TABLE A5:** The ferry route or ferry route subnetwork length, the number of species distributions overlapping with the ferry route or subnetwork, and the climatic bias index.

Ferry route	Ferry length (no. grid cells covered)	Number of species	Climatic bias index
SaAs	404	36	0.3944031
TaGe	278	41	0.17952261
VaSa	218	36	0.42306601
VePa	194	38	1.0530864
CiBa	144	39	0.38805581
AtIz	81	34	1.1377996
ToAl	69	39	0.6424208
ToBas	55	38	0.43982341
KaMi	52	36	1.1377996
ToAj	48	39	0.77694461
NiBas	43	39	0.1230776
LiGA	42	40	1.1377996
ToIR	42	39	0.60432561
SaBas	35	38	0.39494239
NiIR	32	37	0.797778
All ferries	1744	42	0.06914759
Biased subnetwork	513	41	0.3147127
Community subnetwork	1462	42	0.07962026
Environment subnetwork	598	39	0.08448943
